# Editorial: Common themes in drug resistance: from viruses to humans

**DOI:** 10.3389/fmicb.2025.1573798

**Published:** 2025-03-06

**Authors:** Mariana Carmen Chifiriuc, Coralia Bleotu

**Affiliations:** ^1^Faculty of Biology, University of Bucharest, Bucharest, Romania; ^2^Biological Sciences Section, Romanian Academy, Bucharest, Romania; ^3^Department of Immunology, Stefan S. Nicolau Institute of Virology, Bucharest, Romania; ^4^Life, Environmental and Earth Sciences Division, Research Institute of the University of Bucharest—ICUB, University of Bucharest, Bucharest, Romania

**Keywords:** antimicrobial resistance (AMR), HIV resistance, SARS-CoV-2 therapeutics, *Helicobacter pylori* resistance, rhinosinusitis, environmental AMR, tumor cells resistance, dysbiosis-cancer therapy relationship

## Introduction

Drug resistance poses a significant threat to global health, affecting a wide range of biological systems, from viruses and bacteria to human cancer cells. Drug resistance represents one of the greatest threats to human health worldwide. With more than 25,000 deaths every year from infections caused by multidrug resistant (MDR) bacteria (https://health.ec.europa.eu/system/files/2020-01/amr_2017_action-plan_0.pdf), 1,500-million-euro annual costs only in the European Union (EU) and an estimated number of 10 million deaths by 2050 (https://www.jpiamr.eu/resources/what-is-amr/), antimicrobial resistance (AMR) represents a global health priority issue as stated by the World Health Organization (WHO). In addition to AMR, tumor resistance represents an even more threatful issue being responsible for over 90% of cancer deaths (Bukowski et al., 2020). In approximately 50% of cases drug resistance exists even before chemotherapy is initiated (Li and Sperling, 2013).

Understanding the common mechanisms underlying drug resistance and its spread not only in the clinical but also in the environmental sector is crucial for developing effective strategies to combat it. However, the evolutionary processes leading to drug resistance are incompletely known due to the multitude of systems in which they develop (viruses, bacteria, fungi, protozoa, insects, tumor cells, etc.) as well as the driving forces and multiple mechanisms involved in drug resistance.

The aim of this Research Topic was to present the current progress and perspectives regarding drug resistance occurring in different biological systems, from viruses and microorganisms to humans, hoping to provide a worthwhile platform for the future development of new strategies to combat drug resistance. Six papers have been published under this Research Topic, exploring various aspects of drug resistance, from viruses and bacteria to tumoral cells.

## Addressing drug resistance challenges in the most critical viral pathogens in recent history, HIV and SARS-CoV-2

Temereanca and Ruta have performed an in-depth analysis of the challenges posed by HIV drug resistance (e.g., genetic barriers to resistance, role of viral reservoirs, impact of patient adherence on treatment outcomes). The review has also explored novel therapeutic approaches, including the development of drugs targeting different stages of the viral lifecycle and the potential of gene editing technologies to eradicate latent infections and offer additional therapeutic options for patients with multiclass drug-resistant HIV infection (e.g., attachment/post-attachment inhibitors, capsid inhibitors, maturation inhibitors, nucleoside reverse transcriptase translocation inhibitors).

Sǎndulescu et al. review has focused on clinically significant therapeutic developments for the treatment of COVID-19 by providing an in-depth review of molecular mechanisms of action for SARSCoV-2 direct acting and host-directed antivirals and critically analyzing the potential targets that may allow the selection of resistant viral variants. By focusing on host factors essential for viral replication, therapies can potentially reduce the likelihood of resistance development. Various host-targeted approaches, including the use of small molecules and RNA interference, as well as related challenges and opportunities in this emerging field have been highlighted.

## Confronting the escalating crisis of bacterial resistance in clinical and environmental reservoirs

Passing from viral to bacterial resistance, Dascălu et al. have brought into focus the increasing problem of multidrug resistance in *Helicobacter pylori* (HP), a well-known human pathogenic bacterium involved in various gastrointestinal diseases, characterized by chronic progressive gastric inflammation. After updating on the molecular mechanisms underlying resistance, such as point mutations in genes encoding antibiotic targets and the role of efflux pumps, the authors presented the recent developments of novel therapeutic agents to manage resistant *H. pylori* strains, such as vonoprazan-containing triple therapies, quintuple therapies, high-dose dual therapies, and standard triple therapies with probiotics, as well as of anti-Hp vaccines, which could prevent initial Hp colonization or therapeutically, as a possible alternative of or adjunct to the eradication therapy. The paper of Beatrice et al. has addressed the challenges and advancements in the antimicrobial management of complicated rhinosinusitis, a condition characterized by inflammation of the sinuses that extends beyond the sinonasal cavities, potentially leading to severe complications. The authors examine microbial etiology, highlighting the prevalence of multidrug-resistant organisms in these infections. They also discuss the importance of timely diagnosis, appropriate antibiotic selection, and the role of surgical intervention when necessary. Both papers emphasize the need for updated treatment guidelines and personalized treatment strategies to effectively manage drug-resistant cases and prevent further complications.

The global challenge of bacterial resistance in the clinical sector is exacerbated by the environmental antimicrobial resistance (AMR) reservoir, highlighting how interconnected factors in natural ecosystems contribute to the spread and persistence of AMR. In this line with this perspective, Goryluk-Salmonowicz and Popowska paper has focused on the environmental dimensions of AMR, discussing how factors such as the release of antibiotics into natural ecosystems, horizontal gene transfer among microbial communities, and the presence of biofilms contribute to the spread of resistance. The paper also identifies critical knowledge gaps, calling for more research into the environmental reservoirs of resistance and the development of strategies to mitigate their impact. A proper understanding of the role of environmental factors that promote AMR spread requires comprehensive, interdisciplinary approaches contributing to introducing new standards concerning, e.g., the use of antibiotics in veterinary medicine, agriculture or animal farming, and methods of wastewater treatment.

## Unveiling parallels between antimicrobial and cancer drug resistance

In their comprehensive review, Chifiriuc et al. delved into the parallels between AMR and cancer drug resistance. Shared individual and collective mechanisms such as overexpression of efflux pumps, mutations leading to drug target modifications, and the formation of biofilms or tumor microenvironments that protect microbial or tumoral cells, from therapeutic agents have been highlighted. The gut microbiota is influenced by and influences the treatment efficacy and drug toxicity. Chemotherapy is likely to produce *de novo* antimicrobial resistance in gut microbiota by inducing dysbiosis, increasing the horizontal gene transfer and the mutation rates consequently to the bacterial SOS system activation. On the other side, the disruption of commensal gut microbiota and alteration of host physiology might influence both the efficacy of the antitumoral treatments and their toxicity. These strong interconnections and similarities can inform the development of cross-disciplinary strategies to overcome resistance in both infectious diseases and cancer, by using simple and reproducible bacterial models in the development of novel antitumor agents and microbiome-based therapeutic interventions that may be able to correct dysbiosis and thus maximize the treatment efficiency and prevent selection of drug resistance.

Collectively, these contributions underscore the multifaceted nature of drug resistance and the importance of a holistic approach to address it. By understanding the common themes across different systems, we can develop more effective interventions to preserve the efficacy of existing therapies and guide the development of new ones.
